# Effectiveness of Probiotics in Patients With Constipation: A Systematic Review and Meta-Analysis

**DOI:** 10.7759/cureus.52013

**Published:** 2024-01-10

**Authors:** Neyla Garzon Mora, Arturo P Jaramillo

**Affiliations:** 1 Otolaryngology, Universidad Católica de Santiago de Guayaquil, Guayaquil, ECU; 2 General Practice, Universidad Estatal de Guayaquil, Machala, ECU

**Keywords:** efficacy of probiotics, advantages of probiotics, including constipation, functional constipation, probiotics and microbiome

## Abstract

A recent meta-analysis suggests that probiotic supplementation is moderately efficacious in decreasing intestinal transit times compared with control, demonstrating probiotics' potential for treating chronic idiopathic constipation. This decrease in intestinal transit times has been proposed to be attributed to the capacity of probiotics to alter the gastrointestinal microflora, improve intestinal motility, and alter biochemical factors. Therefore, a systematic review and meta-analysis were conducted to estimate the effectiveness of probiotics in patients with constipation. The search was performed using PubMed, PMC, and Medline databases. Relevant data were extracted and assessed for quality using the Cochrane risk of bias assessment tool for randomized clinical trials (RCTs). A random effects model and the I2 statistic were used to estimate the pooled prevalence and explore heterogeneity. Subgroup analyses were conducted based on the experimental group and the placebo group. Sensitivity analysis was performed, and publication bias was explored.

Our meta-analysis assessed probiotics ' efficacy in constipation management by incorporating a sample size comprising 1,243 patients drawn from 10 distinct studies. Subgroup analyses unveiled a heterogeneity of 95%, accompanied by a statistically significant analysis (p-value < 0.05) that unequivocally favored probiotics over placebo for treating constipation. These findings underscore the statistically significant effectiveness of probiotics for individuals with constipation. They support the imperative to fortify this body of evidence through robust, larger-scale RCTs to deepen our understanding of the manifold benefits probiotics confer in nurturing and sustaining optimal gut health.

## Introduction and background

Functional gastrointestinal disorders, such as functional constipation, are frequently observed in medical settings and are one of the most prevalent conditions [1]. Functional constipation is typically diagnosed using the Rome IV diagnostic criteria. This condition is described as a disorder not caused by organic, structural, infectious, or metabolic factors and based on symptoms [2]. Also, this condition does not exhibit any identified structural abnormalities or specific causes [3]. The estimated prevalence of this condition in the adult population is approximately 14%, significantly straining healthcare systems. Work absenteeism, reduced productivity, a decline in quality of life, and increased healthcare costs are some problems arising from constipation and associated digestive discomfort [4]. Physiological, psychological, and sociocultural factors often amplify the impact of patients' symptoms, influencing their daily routines [5].

Functional constipation is thought to have complex and diverse origins and underlying mechanisms. The connection between long-term constipation and imbalances in the gut microbiota has been established through research, indicating that dysbiosis may be an underlying factor in its pathophysiology [6]. The dysbiosis may be influenced by sluggish gastrointestinal transit, potentially limiting gut motility and immune and barrier functions. Nevertheless, the investigation into the correlation between constipation and a disrupted microbiome is currently in its preliminary phases.

Managing functional constipation in a clinical setting poses significant difficulties. Typical methods involve making changes to a patient's diet, adjusting their lifestyle, and utilizing various types of medications such as bulking agents, stool softeners, and osmotic and stimulant laxatives [6]. These therapies frequently encounter limitations such as inconsistent efficacy, varying levels of symptom alleviation, and potential safety issues. In recent times, there has been a growing recognition of the potential benefits of probiotics in regulating intestinal transit time and alleviating symptoms [6]. Probiotics, which are live microorganisms that provide health benefits when consumed in sufficient quantities [7], have a widely documented safety record among the general population.

Guidelines that provide information on safety evaluations for particular strains and their application to at-risk populations have been released [8]. According to a comprehensive analysis of existing research and data, probiotics could improve gut transit time and stool frequency in adults who experience functional constipation. Despite this, the findings of different studies in this area have shown considerable variation [9]. In a separate meta-analysis, it was discovered that the addition of probiotics can decrease the time it takes for food to pass through the intestines. This effect is evident in adults who experience constipation or in older individuals. However, this improvement in intestinal transit time does not always correspond with alleviating symptoms [10-11]. 

Although there is a well-established connection between functional constipation and dysbiosis, few randomized clinical trials (RCTs) have investigated the results of probiotic consumption on microbial alterations and symptom improvements [12]. Preliminary trials have demonstrated that a particular combination of probiotics, such as Lactobacillus acidophilus DDS-1 and Bifidobacterium animalis subsp. lactis UABla-12, may provide relief for individuals suffering from irritable bowel syndrome [12-13]. The primary objective of this systematic review and meta-analysis is to thoroughly assess the latest studies regarding the efficacy of probiotics over placebo in treating patients with constipation.

## Review

Methods

Review Records and Search for Studies

This systematic review adhered to the guidelines of the Preferred Reporting Items for Systematic Reviews and Meta-Analyses (PRISMA) [14]. The article selection process involved two independent researchers conducting comprehensive searches in the PUBMED database. The inclusion criteria encompassed studies published up to August 2023, aligning with the timing of our final article retrieval. Details of the search methodology employed can be found in Table 1.

**Table 1:** Search Strategy for Databases

Search Strategy	Databases Used	Number of Papers Identified
Probiotics AND Constipation AND Bifidobacterium	Pubmed	50
(Probiotics [Title/Abstract]). (Constipation [Title/Abstract]). ((Chronic constipation [Title/Abstract]); OR (Bifidobacterium [Title/Abstract]); AND ((Functional constipation [Title/Abstract]); OR (Chronic constipation [Title/Abstract]).	Pubmed Central	568
"Probiotics [tw]" AND "Constipation [tiab]" AND "Bifidobacterium[all]"	Medline	1

Inclusion and Exclusion Criteria

Two independent authors used the Covidence software to screen the search results obtained from two databases following pre-established inclusion and exclusion criteria, as shown in Table 2.

**Table 2:** Inclusion and exclusion criteria

Inclusion criteria	Exclusion criteria
Free, full text about probiotic supplementation	Absence of an abstract of the full paper
Articles from the past 10 years	Articles from 2012 and below
English-language articles	Non-English studies
Prospective or retrospective studies.	Case reports
Human trials	Animal trials

Data Extraction

Several key observations have been made from an in-depth examination of the studies in question. These include the design of each study, the count of participants administered probiotics, the characteristics of the placebo group, and the results noted in both the experimental and placebo cohorts.

Risk of Bias Assessment

To assess potential biases in the studies selected for our research, we employed the Cochrane risk of bias tool, specifically designed for randomized controlled trials (RCTs). This tool is widely recognized for its effectiveness in evaluating the quality of case-series studies [15]. Each study's risk of bias was independently appraised by two reviewers, with any differences in their evaluations being reconciled through comprehensive discussions.

Statistical Analysis

RevMan version 5.4 (2020; The Cochrane Collaboration, The Nordic Cochrane Centre, Copenhagen, Denmark) was utilized for all statistical analyses. The mean difference with 95% confidence intervals was used to present the trial results, and an odds ratio effects model was used to pool them. The method outlined by Mantel-Haenszel et al. was used to calculate the standard deviations or standard errors if they weren't reported in the trial. Given the possible high variance between studies due to different study designs and populations, we used a fixed-effect model rather than a random-effect model.

Forest plots were generated to evaluate the pooling results visually. Any differences between the subgroups were found using the chi-square test. Higgins I2 was used to measure study heterogeneity. A visual inspection of the funnel plot was utilized to assess publication bias, and p<0.05 was used to determine statistical significance. 

Results

**Figure 1:**
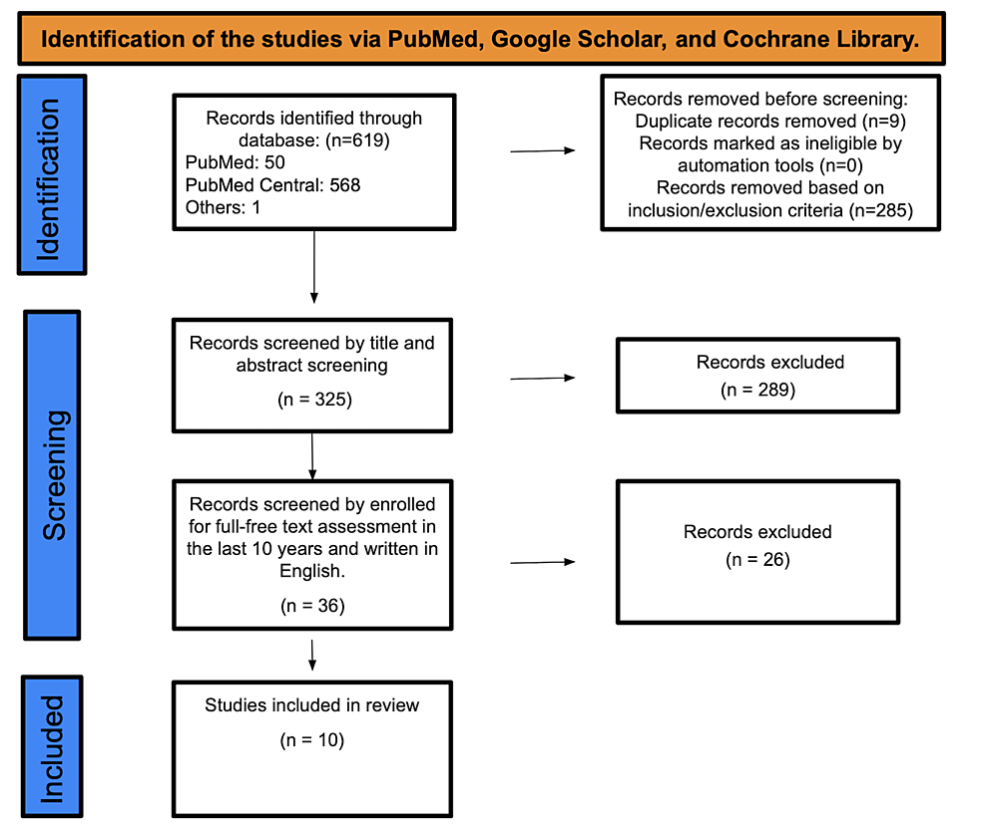
PRISMA diagram

A total of 619 studies were found after searching PubMed, Medline, and PMC. Two hundred eighty-five were marked as ineligible based on inclusion and exclusion criteria, and 9 duplicate studies were removed. A total of 325 studies underwent title and abstract screening, with 289 papers being discarded since they were not related to the purpose of our study. The remaining 36 papers were chosen based on their content in English and full-free text evaluation in the previous ten years, eliminating 26 studies; only 10 were enlisted for the final data collection-identification of studies via databases and registers (Figure 1). See Table 3 for an in-depth description of the articles we decided to use.

**Table 3:** Table of data extraction RCT: Randomized clinical trial; CRC: colon rectal cancer; BMI: body mass index; TWK10: Lactobacillus plantarum

Author	Year of publication	Study design	Primary research	Outcome evaluation
Takeda et al. [16]	2023	RCT	Participants were divided into two groups for the study: the treatment group (n = 39) and the placebo group (n = 41).	The main outcome measure did not show statistical significance (P = 0.074). However, there was a significant improvement (P < 0.01) observed in the experimental group compared to baseline in the 1 month. Conversely, no significant changes were observed in the placebo group.
Ma et al. [17]	2023	RCT	A 1 month and 12 days study was conducted to examine the impact of probiotics on relieving CC. The trial followed a randomized, double-blind, and placebo-controlled design.	The consumption of probiotics resulted in a notable enhancement in the average number of CSBMs and SBMs per week.
Lai et al. [18]	2023	RCT	A study was conducted over a period of 1 month, involving 250 adults who were diagnosed with FC. The trial was designed to be double-blinded and randomized, with a placebo control group.	Research has indicated that the consumption of dietary fibers or probiotics might be effective in alleviating constipation by promoting changes in gut microbiota that are specifically associated with relieving hard stool.
Šola et al. [19]	2022	RCT	The study included a total of 60 participants, consisting of 42 females and 18 males. All participants had FC and met the necessary eligibility criteria.	Participants were randomly assigned to receive either a probiotic mixture (N = 28) or a placebo (N = 32) for a duration of 3 months, in addition to their regular diet and medications.
Mitelmão et al. [20]	2022	RCT	Two formulations were developed in the form of an oral sachet containing probiotics, and their efficacy and safety were evaluated in adults with functional constipation.	The two probiotic cocktails were effective in improving the symptoms of FC
Martoni et al. [13]	2019	RCT	In total, a group of 94 adults who were otherwise in good health, and experiencing symptoms of FC, were randomly selected for the ITT.	The PAC-SYM questionnaire did not show any notable differences between the groups, although there were significant differences within each group (P < 0.001) throughout the duration of the study.
Ibarra et al. [21]	2018	RCT	A group of 228 adults who were diagnosed with FC based on the Rome III criteria were randomly assigned to participate in trials that involved the use of placebos.	No significant differences were found in the primary or secondary outcomes between the interventions.
Moreira et al. [22]	2017	RCT	A study was conducted involving 49 women diagnosed with constipation based on the ROME III criteria. The trial was randomized and double-blind in nature.	The findings indicate that the ingestion of milk led to the alleviation of constipation symptoms, irrespective of the type of probiotic culture used.
Ojetti et al. [23]	2014	RCT	The RCT included a sample of 40 adults with an average age of 35 years who were diagnosed with FC based on the Rome III criteria.	In previous studies conducted on children, it has been shown that L. reuteri is more beneficial than a placebo in enhancing the frequency of bowel movements in adult patients with FC. However, it appears that L. reuteri does not have an impact on stool consistency.
Jayasimhan et al. [24]	2013	RCT	A group of 120 adults suffering from constipation, who were diagnosed using the Rome III criteria, were randomly assigned to receive either a microbial cell preparation or a placebo. The participants were instructed to consume their assigned treatment twice daily.	The use of microbial cell preparation has been found to be beneficial in enhancing both the frequency and consistency of stool.

Quality Assessment

In our systematic review of literature and meta-analysis, we undertook a stringent, multidimensional approach to quality assessment, heightening the credibility and reliability of our findings. We rigorously applied the Cochrane risk of bias assessment tool to evaluate the methodological rigor of the included RCTs and detect potential biases.

After assessing 10 randomized controlled trials (RCTs) for quality, we attributed six "+" to four of them and seven "+" to six. We considered these studies high quality and decided to include them in our systematic review. The results are exposed in Table 4.

**Table 4:** Quality assessment of RCTs

Studies	Random sequence generation (selection bias)	Allocation concealment (selection bias)	Blinding of participants	Blinding of personnel/care providers (performance bias)	Blinding of outcome assessor (detection bias)	Incomplete outcome data (attrition bias)	Selective reporting (reporting bias)	Other biases	Overall
Author	+	+	+	+	+	+	+	-	7/8
Takeda et al. [16]	+	+	+	+	+	+	+	-	7/8
Ma et al. [17]	+	+	+	+	+	+	+	-	7/8
Lai et al. [18]	+	+	+	+	+	+	+	-	7/8
Šola et al. [19]	+	+	+	+	+	+	+	-	7/8
Mitelmão et al. [20]	+	+	+	+	?	+	+	-	6/8
Martoni et al. [13]	+	+	+	+	-	+	+	-	7/8
Ibarra et al. [21]	+	+	+	+	?	+	+	-	6/8
Moreira et al. [22]	+	+	+	+	?	+	+	-	6/8
Ojetti et al. [23]	+	+	+	+	?	+	+	-	6/8
Jayasimhan et al. [24]	+	+	+	+	?	+	+	-	7/8

Meta-Analysis of Outcomes

**Figure 2:**
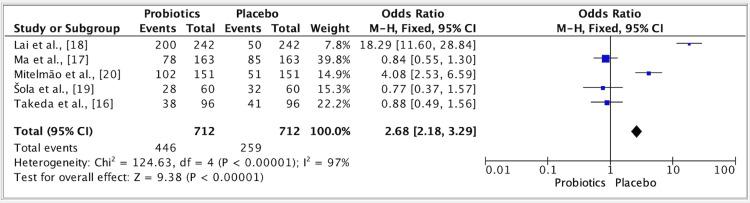
Forest plot for studies about the efficacy of  probiotics vs. placebo groups References: [16-20]

The results of five studies showed an odd ratio of 2.68 for the efficacy of probiotics vs. placebo groups. The odd ratio was 2.68 (fixed effect, 95%). The CI was 2.18-3.29, and the P value was <0.01, and the heterogeneity (I2) was 97% (Figure 2).

**Figure 3:**
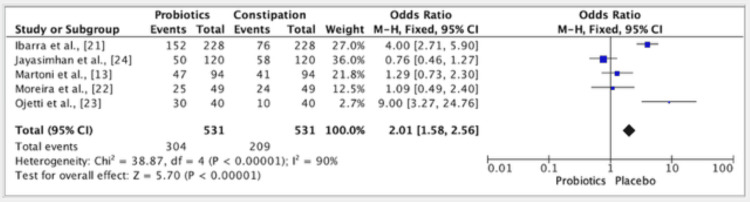
A forest plot for studies about the efficacy of probiotics vs. placebo groups References: [13,21-24]

The results of five studies showed an odd ratio of 2.01 in the efficacy of probiotics vs. placebo groups. The odds ratio was 2.01 (fixed effect, 95%). The CI was 1.58-2.56, the P value was 0.001, and the heterogeneity (I2) was 90% (Figure 3).

**Figure 4:**
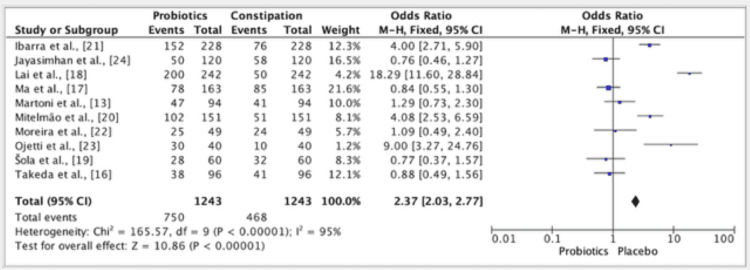
Forest plot for studies about the overall efficacy of  probiotics vs. placebo groups References: [13,16-24]

**Figure 5:**
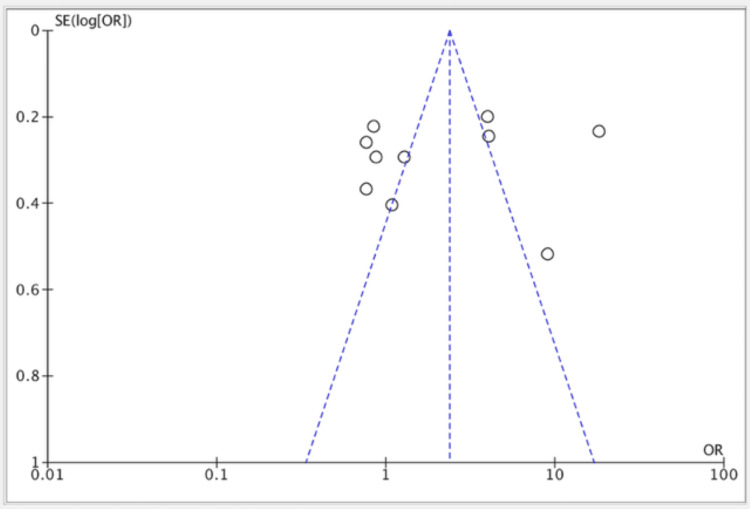
Funnel plot for all included studies about the efficacy of probiotics vs. placebo groups References: [13,16-24]

The results of ten studies showed an odd ratio of 2.37 in the efficacy of probiotics vs. placebo groups. The odds ratio was 2.37 (fixed effect, 95%). The CI was 2.03-2.77, the P value was <0.01, and the heterogeneity (I2) was 95% (Figure 4). Publication bias was seen in all of the studies (Figure 5).

Discussion

Functional constipation and chronic constipation, distinguished by their characteristic hard, infrequent, or incomplete bowel movements and lack of an organic basis, affect roughly 10.1% of the adult population worldwide, as per the Rome IV criteria. These conditions significantly diminish life quality and increase healthcare costs. Addressing functional constipation is critical, especially in demographics experiencing rapid aging, such as those in China. Our review synthesizes data from various studies to offer a detailed view of the current research. We divide our discussion into two sections: one focusing on research showing significant results with probiotic use for constipation treatment and the other on studies where results were not statistically significant.

The RCT conducted by Takeda et al. assessed BB536's impact on constipation and abdominal symptoms in older individuals with chronic constipation, employing the constipation scoring system and the gastrointestinal reflux disease symptom frequency scale [16]. Their findings showed notable improvements in bowel movement regularity and upper abdominal symptoms within four weeks of starting BB536, surpassing the results seen in the placebo group. Additionally, symptom score enhancements were present four weeks posttreatment, possibly due to the probiotics positively altering the intestinal environment [16]. Ma et al.'s research revealed that P9 administration significantly alleviated chronic constipation in adults, enhancing life quality aspects, including increasing the average weekly frequency of complete spontaneous bowel movements and spontaneous bowel movements and decreasing worries and concerns [17]. Lai et al.'s RCT highlighted the role of dietary fibers and probiotics in easing hard stools in individuals with functional constipation [18]. Their study indicated evolving microbiota features and identified specific baseline microbial profiles that could predict responsiveness to the interventions, hinting at a link between microbiota changes and symptom relief. They found that a daily intake of either 5 g of psyllium husk or a combination of 2 g of psyllium husk and 5 g of wheat bran with oligosaccharide over four weeks effectively eased hard stools [18]. Daily supplementation with Bifidobacterium animalis subsp. lactis and Lacticaseibacillus rhamnosus also showed positive results. A prior meta-analysis suggested Bifidobacterium animalis subsp. lactis significantly enhanced the Bristol stool scale score, though this effect was not observed with other strains [18].

Mitelmo et al.'s RCT demonstrated that probiotic blends containing three or eight strains effectively improved functional constipation symptoms, increasing evacuation frequency and stool quality from the first week of treatment [20]. They highlighted the cost-effectiveness of prebiotics over probiotics, though probiotics' diverse health benefits were evident, including gut health maintenance, pathogenic bacteria inhibition, and immune system enhancement [20]. Ojetti et al.'s study also confirmed the efficacy of L. reuteri in increasing bowel movement frequency in adults with chronic functional constipation [23]. The sustained positive effects over one month suggest this strain's potential long-term benefits and safety. Jayasimhan et al.'s RCT noted a higher stool frequency in the treatment group with less straining, a decreased sensation of incomplete evacuation, and improved stool consistency compared to the placebo group [24]. However, differences in anorectal blockage sensations and the need for manual defecation maneuvers were not statistically significant [24].

In the RCT by Šola et al., the probiotic and placebo groups showed similar total stool counts over 91 days, but the probiotic group had consistently higher counts from the first week, reaching statistical significance after 71 days [19]. Martoni et al. reported no significant difference in symptoms between groups but noted an earlier bowel function modulation and microbiota shift toward a more fibrolytic profile in the probiotic group [13]. Unlike the placebo group, participants receiving probiotics showed a significant increase in genome equivalents for evaluated strains, with no significant disruption in overall microbiota composition [13]. Ibarra et al. did not find significant differences in primary or secondary outcomes, including colonic transit time and weekly bowel movement frequency [21]. All participants experienced similar placebo effects regarding relief from constipation, with more improvements than deteriorations noted, although the placebo group alone showed significant improvement over baseline in the intention-to-treat population [21].

Our research encompassed a methodical investigation, wherein we systematically combed through databases to pinpoint studies contrasting the efficacy of probiotics versus placebos in individuals with constipation. Our data compilation involved a cohort of 1,243 patients extracted from 10 distinct studies. We adhered to a meticulously designed framework, encompassing a standardized search strategy, comprehensive evaluation for publication bias, sensitivity analysis, and in-depth scrutiny of heterogeneity through subgroup analysis. These methodological strides played a pivotal role in shaping the conclusive findings derived from our study.

## Conclusions

Our comprehensive analysis has yielded significant insights into the role of probiotics in managing gastrointestinal disorders. Among the 10 peer-reviewed studies analyzed, a substantial majority (70%) reported positive outcomes in treating functional and chronic constipation. These findings are pivotal, as they highlight the potential of probiotics to not only enhance bowel movement frequency and caliber but also to significantly improve the overall quality of life for patients suffering from these conditions. Furthermore, our study observed the notable efficacy of probiotics in ameliorating upper gastrointestinal symptoms, including gastroesophageal reflux, which adds a new dimension to the therapeutic scope of probiotics. Intriguingly, the synergistic effect of probiotics when combined with dietary fibers such as psyllium husk or wheat bran was evident, suggesting an enhanced benefit to gastrointestinal health.

Although these results are promising, it is critical to approach them with a balanced perspective. The variability in probiotic strains, dosages, and study designs necessitates further investigation to establish probiotics' efficacy, safety, and long-term benefits in gastrointestinal health conclusively. Therefore, we advocate for more rigorous, large-scale RCTs to validate these preliminary findings and explore the full potential of probiotics as a therapeutic modality in gastrointestinal disorders.
